# Dying to help: Fatal bystander rescues in Australian coastal environments

**DOI:** 10.1371/journal.pone.0238317

**Published:** 2020-09-16

**Authors:** Jasmin C. Lawes, Eveline J. T. Rijksen, Robert W. Brander, Richard C. Franklin, Shane Daw

**Affiliations:** 1 Surf Life Saving Australia, Sydney, NSW, Australia; 2 School of Biological, Earth and Environmental Sciences, UNSW Sydney, Sydney, NSW, Australia; 3 College of Public Health, Medical and Veterinary Sciences, James Cook University, Townsville, Qld., Australia; South China University of Technology, CHINA

## Abstract

Bystanders who drown during a rescue attempt in aquatic waterways are becoming an increasingly important issue within drowning prevention. In the Australian context, the majority of these incidents occur in coastal water ways. This study documents and characterizes bystander rescuer fatalities within Australian coastal waterways that occurred between 1 July 2004 and 30 June 2019 in order to provide suggestions for future public safety interventions involving bystander rescuers. Data was sourced through Surf Life Saving Australia’s (SLSA) Coastal Fatality Database, which collates information from multiple sources. Sixty-seven bystander rescuer fatalities in coastal waterways were reported during the 15-year period, an average of 4.5 per year, which is a significant proportion of the five fatalities previously reported across all Australian waterways. The majority of coastal bystander rescuer fatality incidents occurred in the state of New South Wales (49%), at beaches (64%), in regional or remote areas (71%), more than 1 km from the nearest lifesaving service (78%), during summer (45%), in the afternoon (72%), in the presence of rip currents (73%), and did not involve the use of flotation devices to assist rescue (97%). The majority of coastal bystander rescuer victims were Australian residents (88%) born in Australia/Oceania (68%), males (81%), aged between 30–44 years old (36%), visitors to the location (55%), either family (69%) or friends (15%) of the rescuee(s), and were attempting to rescue someone younger than 18 years old (64%). Our results suggest future safety intervention approaches should target males, parents and carers visiting beach locations in regional locations during holiday times and should focus on the importance of flotation devices when enacting a rescue and further educating visitors about the rip current hazard. Future research should examine the psychology of bystander rescue situations and evaluate the effectiveness of different safety intervention approaches.

## Introduction

With an estimated 360,000 annual drowning deaths worldwide, drowning is the third leading cause of unintentional injury death globally [1] and is also a multi-faceted and complex issue involving a range of social, cultural and environmental determinant factors [2]. The World Health Organisation [3, 4] has identified six evidence-based interventions to approach the problem of drowning prevention, one of which is to ‘train bystanders in safe rescue and resuscitation’. In this context, a ‘bystander’ is defined as any member of the public, be they family, friend or stranger, who offers assistance (by attempting a rescue) to someone in distress [5, 6]. The issue of aquatic rescues undertaken by bystanders is significant in drowning prevention for two fundamentally important reasons. First, in the absence of lifeguards, bystanders often represent the only form of assistance to those in distress, or may be the nearest first responders in the presence of lifeguards, therefore potentially providing a valuable (and unofficial) lifesaving service [7–11]. Second, it is unfortunately not uncommon for the bystander themselves to drown while attempting a rescue [11]. This is due to a number of factors including impulsive and emotive reactions of the rescuer and a lack of necessary skills to assess the situation, associated hazards, and ability to carry out a successful rescue and resuscitation [5, 8, 12–15].

The World Health Organisation [4] also suggest that the implementation of any drowning prevention intervention should begin with a situational assessment to establish key facts that are essential to proper prioritization and planning of the intervention, the first component of which is to review available data. In this regard, the topic of bystander rescues has only received modest attention in the drowning prevention literature and while fatal bystander rescues undoubtedly occur at an international scale, documentation is somewhat limited, largely due to a lack of accurate, reliable and systematic incident reporting and data availability [5, 13, 16–19]. Recent studies by Brander, Warton [10] and Franklin, Peden [11] summarised the characteristics and global extent of the problem, while focussing on the Australian context, and used quantitative surveys to describe characteristics and experiences of bystander rescues and rescuers in a range of different Australian aquatic environments. Of note, Franklin, Peden [11] found that 23% of surveyed respondents had previously undertaken a rescue in an aquatic environment and reported that 51 people drowned while performing an aquatic rescue in Australia between 2006–2015, an average of 5 per year. Brander, Warton [10] found that 70% of surveyed bystander rescuers felt they had saved a life by performing a rescue.

Australia benefits from the existence of reliable and increasingly long-term databases related to drowning and coastal fatalities [11]. Surf Life Saving Australia (SLSA), Australia’s main coastal water safety, drowning prevention, and rescue authority curates the Coastal Fatality Database, which collates all available information on drowning deaths and other fatalities that occur across the Australian coast. The primary aim of this study is to use this Database to provide quantitative information on the occurrence and characteristics of bystander rescue related drowning in Australia’s coastal waters.

The focus on coastal incidents provides a valuable extension of the studies by Brander, Warton [10] and Franklin, Peden [11] and attempts to provide a data review that is a critical starting point for establishing a situational assessment for any future intervention relating to bystander rescues in coastal waters. A secondary aim is to examine known characteristics of bystander rescue fatalities to provide further insights into contributory factors leading to these incidents. It is hoped that this information can be used to guide and assist any future prevention and education strategies related to the issue of bystander rescues.

## Materials and methods

Data for coastal bystander rescuer fatalities for a 15-year period from 1 July 2004 to 30 June 2019 was sourced through the SLSA Coastal Fatality Database. This Database collates information from the media, the Australian National Coronial Information System (NCIS) and SLSA’s SurfGuard Incident Report Database (IRD).

The National Coronial Information System is an online repository of coronial data from Australia and New Zealand. Information from the NCIS database provides the backbone of the Coastal Fatality Database and is used to proof information from other sources. The NCIS provides clear, high quality data that can assist with preventing future deaths. Any information gathered during a coronial investigation regarding the circumstances around or leading to any non-natural deaths or unexpected deaths in Australia, are gathered, quality assured and collated into a database repository managed by the NCIS. Cases in the NCIS remain open (i.e. under investigation) until the Coroner makes a ruling regarding the cause of death and the case is closed. At time of analysis, 82% of fatal bystander rescue cases (n = 55) were closed. For open cases, data is correct as of 14 October 2019.

The SurfGuard IRD is a web-based portal used by SLSA services to electronically record incident reports. Reports are required for all incidents where medical treatment is administered and include search and rescue operations. Reports are regularly uploaded to the SurfGuard database by surf lifesaving club, branch, or state personnel.

Google alerts track and monitor related news published in newspapers, magazines, journals, television, radio, internet and social media. Daily reports are produced that include media relating to Australian coastal incidents and hazards.

This study was conducted with ethics approval from the Department of Justice and Regulation Human Research Ethics Committee (JHREC; CF/07/13632; CF/10/25053; CF/16/17314).

### Data inclusions

Data used in this study relates to unintentional fatalities resulting from the attempted rescue of someone else in distress in Australian coastal waters. Cases were included where the primary medical cause of death was drowning, or if drowning was a contributing factor. Incidents where the primary cause of death was not drowning (i.e. medical episodes such as cardiac events), but the incident occurred while attempting a rescue in the water, were also included. ‘Coastal waters’ are defined based on the New Zealand Resource Management Amendment Act 1993 as ‘the foreshore, seabed, coastal water and air space above a large body of water (harbour/bay/inlet), including up to three nautical miles offshore and of which the landward boundary is the line of mean high water, except where that line crosses a river or inlet, the landward boundary at that point shall be the point upstream that is calculated by multiplying the width of the river or inlet mouth by five’ [20; Adopted from the Resource Management Amendment Act 1993 –New Zealand].

Data variables collected relating to both the incident and the victim are described in Table 1. The ‘remoteness classification’ of an incident location was coded to the Australian Statistical Geographic Standard Remoteness Areas (Table 1) determined for each location by using the Health Workforce Locator website [21]. The presence of rip currents, which are strong, narrow seaward flowing currents that are recognised as being a major factor involved in beach-related drowning in Australia [22, 23], was classified ‘yes’ or ‘no’ depending on if a rip current was reported to be present in the incident or not (Table 1). The use of any flotation device (e.g. surfboard, a lifejacket or any other buoyant object) by the bystander rescuer was classified as ‘yes’ if used and ‘no’ if not used. The distance from nearest surf lifesaving club or lifeguard service was calculated using the SLSA Beachsafe website (https://beachsafe.org.au).

**Table 1:** Variable categorisation and definitions.

Incident-related Variables		Definition	Victim-related Variables		Definition
	Categories			Categories	
**State**			**Gender**		
	NSW	Australian state or territory in which the incident occurred		Male	Gender of bystander (victim)
	VIC			Female	
	QLD		**Age (years)**		
	WA			0–17	Age of bystander (victim)
	SA			18–29	
	NT			30–44	
	TAS			45–59	
**Remoteness**				60+	
	Major Cities	Remoteness of incident as determined using the Health Workforce Locator (REF)	**Activity**		
	Inner Regional			Swimming/Wading	Activity or primary reason bystander (victim) visited incident location
	Outer Regional			Rock Fishing	
	Remote			Boating	
	Very Remote			Snorkelling/Scuba	
**Season**				Land-based Fishing	
	Summer (Dec-Feb)	Season in which incident occurred		Other	
	Autumn (Mar-May)		**Visitor status**		
	Winter (Jun-Aug)			Local (<10km)	Visitor status of bystander (victim), relative to incident location
	Spring (Sep-Nov)			Resident (10-50km)	
**Time of day**				Intrastate (>50km)	
	Morning (6:01–12:00h)	Time incident occurred		Interstate	
	Afternoon (12:01–18:00h)			International	
	Evening (18:01–0:00h)			Unknown *	
	Night (0:01–6:00h)		**Toxicology**		
**Distance from Surf Lifesaving Services**				Alcohol/Drugs	Alcohol/drug contribution to death (coroner report)
	< 1 km	Distance incident occurred from Surf Lifesaving Service		None	
	1–5 km		**Birth continent**		
	> 5 km			Australia/Oceania	Continent where bystander (victim) was born
**Location**				Asia	
	Beach	Coastal location type where incident occurred		Europe	
	Rocks/Cliff			Unknown *	
	Offshore		**Continent of residence **		
	Bay			Australia	Continent where bystander (victim) resided
	Marina/Jetty			Asia	
**Rip current presence**				Europe	
	Yes	Presence of a rip current reported at time of incident		Unknown *	
	No		**Rescuee relationship**		
	Unknown			Family/Spouse	Relationship or level of familiarity between bystander (victim) and rescuee
**Flotation device used**				Friend	
	Yes	Use of flotation device by bystander (victim)		Stranger	
	No			Other	
**Day of week**			**Rescuee age **		
	Weekend day	Whether incident occurred on weekday or weekend		Child (<18)	Age of rescuee
	Weekday			Adult (18+)	
				Other/Unknown *	

* Shows variables that were excluded from analyses.

‘Visitor status’ was calculated relative to incident location using Google Maps to determine the distance in kilometres between the incident postcode and the bystander victims’ residential postcodes (Table 1). Bystander victim toxicology was collated from coronial toxicological analyses and was coded as ‘alcohol’ if blood alcohol content of the rescuer exceeded 0.05% (the legal limit for driving in Australia); ‘drugs’ if drugs, whether illicit, prescription, over-the-counter or a combination, were present in the rescuer and the coroner ruled that it contributed to the death; ‘alcohol and drugs’ if both criteria applied; and ‘none’ if rescuer blood alcohol content was under 0.05% and no drugs were found to have contributed to the death. Since numbers of incidents involving alcohol and drugs were low, they were combined into a single category for analyses (alcohol/drugs; Table 1). Rescuee age was also coded as child or adult in alignment with Australian eligibility to vote (18 years old or over).

### Statistical analyses

Data coding and analysis were conducted in SPSS Statistics v26.0 (SPSS IBM Corp. ©). Data analyses were performed with unknown variables excluded. Non-parametric tests were conducted on population-based variables e.g. age, gender, state, and remoteness were performed using the mean proportional basis of the Australian population as expected outcome numbers (ABS). Analyses of incident-related or environmental variables e.g. time of day, season, rip current presence, incident location, distance from surf lifesaving services, toxicology and activity undertaken prior to rescue attempt analyses were compared against proportions of unintentional coastal drowning deaths that were not bystander rescue-related (referred to herein as *non-rescue incidents*) collated in the SLSA Coastal Fatality Database as expected outcome numbers (drowning deaths were selected over other fatalities as it was considered a more comparable cohort). For variables that were not population-based or incident-related, binomial and one-sample chi-square analyses were used to compare rescuee relationship categories, rescue age and whether a flotation device was used (Table 1). Binomial analyses were used when there were two categories and chi-square was used when there were three or more subgroups within a category. Groups with small counts (i.e.< 5) were consolidated where possible and Fisher’s Exact Test was used for more stable estimates. Statistical significance was accepted for p-values <0.05.

## Results

**Fig 1:**
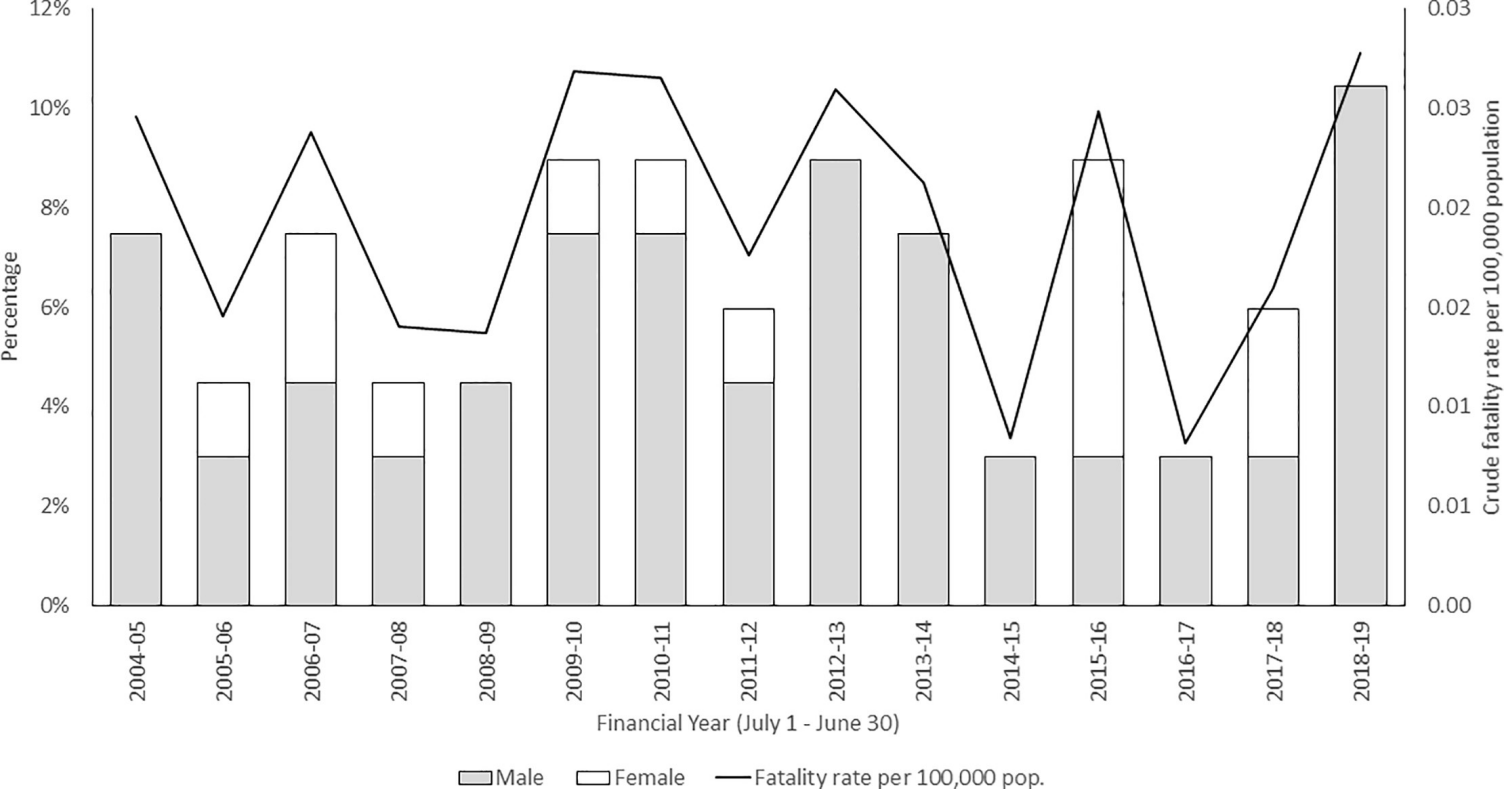
Annual percentage of coastal fatal bystander rescues in Australia by gender per financial year (July 1 to June 30) with crude fatality rate per 100,000 population between 1^st^ July 2004 and 30^th^ June 2019.

During the 15-year period between 1 July 2004 and 30 June 2019 there were 67 fatal bystander rescuer incidents in Australian coastal waters (Fig 1). Of note, 18% (n = 12) of these incidents remained under investigation by coroners at time of analysis (14 October 2019). Sixty-five incidents were drowning deaths while two were fatalities due to medical episodes (e.g. heart attack) that occurred while performing a rescue. This equates to an average of 4.5 coastal bystander rescuer deaths per year, a crude fatality rate of 0.02 deaths per 100,000 population. As evident from Fig 1, there is no obvious temporal trend in the data. Incidents did not occur proportionally across Australia (*Χ*^*2*^ = 17.994; p = 0.013; Table 2) with almost half (49%, n = 33) occurring within the state of New South Wales, followed by Victoria (16%, n = 11) and Queensland (13%, n = 9).

**Table 2:** Descriptive analyses of fatal coastal bystander rescue incidents.

Incident-related Variables		Annual average (%)	Expected Proportion (%)	Test statistic (p-value)	Victim-related Variables		Annual average (%)	Expected Proportion (%)	Test statistic (p-value)
**State** _**a**_					**Gender** _**a**_				
** **	NSW	2.2 (49)	49	***X***^***2***^ **= 17.994 (p = 0.013)**^**E**^		Male	3.6 (81)	50	***Χ***^***2***^ **= 25.090 (p < 0.001)**
** **	VIC	0.73 (16)	16			Female	0.87 (19)	50	
** **	QLD	0.6 (13)	13		**Age** _**a**_				
** **	WA	0.33 (7)	7			0–17	0.2 (4)	23	***X***^***2***^ **= 22.644 (p < 0.001)**^**E**^
** **	SA	0.27 (6)	6			18–29	1.07 (24)	17	
** **	NT	0.2 (4)	4			30–44	1.6 (36)	21	
** **	TAS	0.13 (3)	3			45–59	1.13 (25)	19	
**Remoteness** _**a**_						60+	0.47 (10)	19	
** **	Major Cities	1.27 (28)	70	***X***^***2***^ **= 61.654 (p < 0.001)**^**E**^	**Activity before rescue attempt** _**b**_				
** **	Inner Regional	1.93 (43)	19			Swimming/Wading	3.13 (70)	36	***X***^***2***^ **= 54.078 (p < 0.001)**^**E**^
** **	Outer Regional	0.87 (19)	9			Rock Fishing	0.33 (7)	13	
** **	Remote/Very Remote	0.4 (9)	2			Boating	0.27 (6)	21	
** **						Snorkelling/Scuba	0.27 (6)	12	
**Season** _**b**_						Land-based Fishing	0.27 (6)	1	
** **	Summer	2 (45)	45	*X*^*2*^ = 6.232 (p = 0.101)^**E**^		Other	0.2 (4)	16	
** **	Autumn	1.4 (31)	34		**Visitor status** _**b**_				
** **	Winter	0.2 (4)	4			Local	1.47 (35)	30	***X***^***2***^ **= 10.470 (p = 0.033)**
** **	Spring	0.87 (19)	19			Resident	0.4 (10)	26	
**Time of day** _**b**_						Intrastate	1.27 (31)	27	
** **	Morning	0.67 (15)	34	***X***^***2***^ **= 10.869 (p = 0.004)**		Interstate	0.53 (13)	7	
** **	Afternoon	3.2 (72)	54			International	0.47 (11)	10	
** **	Evening	0.6 (13)	12			Unknown	0.33 (*)	*	
** **	Night	0 (*)			**Toxicology** _**b**_				
**Distance from Surf Lifesaving Services** _**b**_						None	3.8 (85)	77	*X*^*2*^ = 2.250
** **	< 1 km	1 (22)	33	*X*^*2*^ = 3.563 (p = 0.168)		Alcohol/Drugs	0.67 (15)	23	(p = 0.134)
** **	1–5 km	1.33 (30)	23		**Birth continent** _**a**_				
** **	> 5 km	2.13 (48)	44			Australia/Oceania	2.73 (68)	76	***X***^**2**^ **= 64.639 (p < 0.015)**
**Location** _**b**_						Asia	0.87 (22)	10	
** **	Beach	2.87 (64)	50	***X***^***2***^ **= 9.421 (p = 0.024)**^**E**^		Europe	0.4 (10)	11	
** **	Rocks/Cliff	1 (22)	20			Unknown	0.47 (*)	*	
** **	Offshore	0.27 (6)	18		**Continent of residence** _**a**_				
** **	Bay/ Marina /Jetty	0.33 (8)	12			Australia	3.87 (88)	78	*X*^2^ = 1.078 (p = 0.583)^**E**^
						Asia	0.4 (9)	13	
**Rip presence** _**b**_						Europe	0.27 (6)	6	
** **	Yes	3.07 (73)	23	***X***^***2***^ **= 88.793 (p < 0.001)**		Unknown	0.07 (*)	*	
** **	No	1.13 (27)	77		**Rescuee relationship** _**d**_				
** **	Unknown	0.27 (*)	*			Family	3.07 (69)	25	***X***^***2***^ **= 69.537**
**Flotation device used** _**c**_						Friend	0.67 (15)	25	**(p < 0.001)**
	Yes	0.13 (3)	50	**z = 64.000 (p < 0.001)**		Stranger	0.4 (9)	25	
	No	4.33 (97)	50			Other	0.33 (7)	25	
**Day of week** _**d**_					**Rescuee age** _**c**_				
	Weekend day	2.2 (49)	29	***X***^***2***^ **= 13.348 (p < 0.001)**		Child (<18)	2.73 (64)	50	**z = 41.00 (p = 0.034)**
	Weekday	2.27 (51)	71			Adult (18+)	1.53 (36)	50	
						Other/Unknown	0.2 (*)	*	

* Shows excluded variables, **bold text** denote significance, subscripts denote analysis method. Annual average is presented for ethical requirements.

_a_ Proportions compared with mean population proportions (2004–19)

_b_ proportions compared against non-rescue incidents (2004–19)

_c_ one-sample binomial analysis

_d_ one-sample chi-square analysis

^E^ shows p-value reported is Fishers Exact Test.

**Fig 2:**
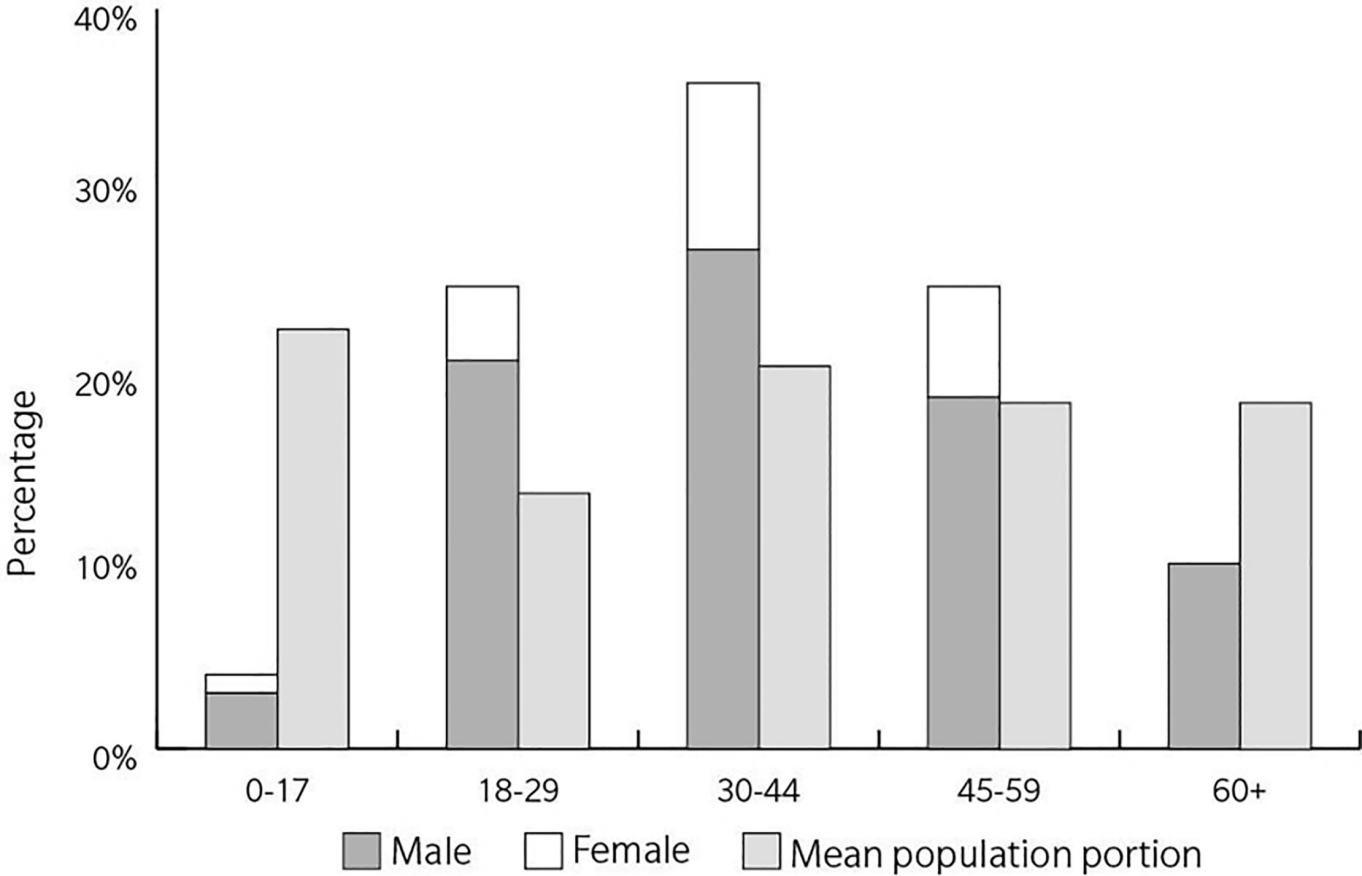
Bystander rescue victim age proportions by gender and compared to mean Australian population proportions (2004–19).

Males were the dominant gender involved in bystander drowning deaths (81%, n = 54; *Χ*^*2*^ = 25.090; p < 0.001; Table 2). Nearly two-thirds of coastal bystander rescue victims were between 18–44 years of age (60%, n = 40) and 91% were under 60 years of age (Fig 2). In terms of age, the 30–44 age bracket was associated with the highest number of fatalities with the lowest being within the 0–17 year old age bracket (Fig 2). Of note, there were no fatalities for females aged 60 and over compared to a total of 7 males in this age group (Fig 2). In general, age groups of bystander victims differ from expected mean Australian population proportions (*Χ*^*2*^ = 22.644; p < 0.001) where 18–44 year old victims were higher and 0–17 and 60+ age groups were lower than expected.

**Fig 3:**
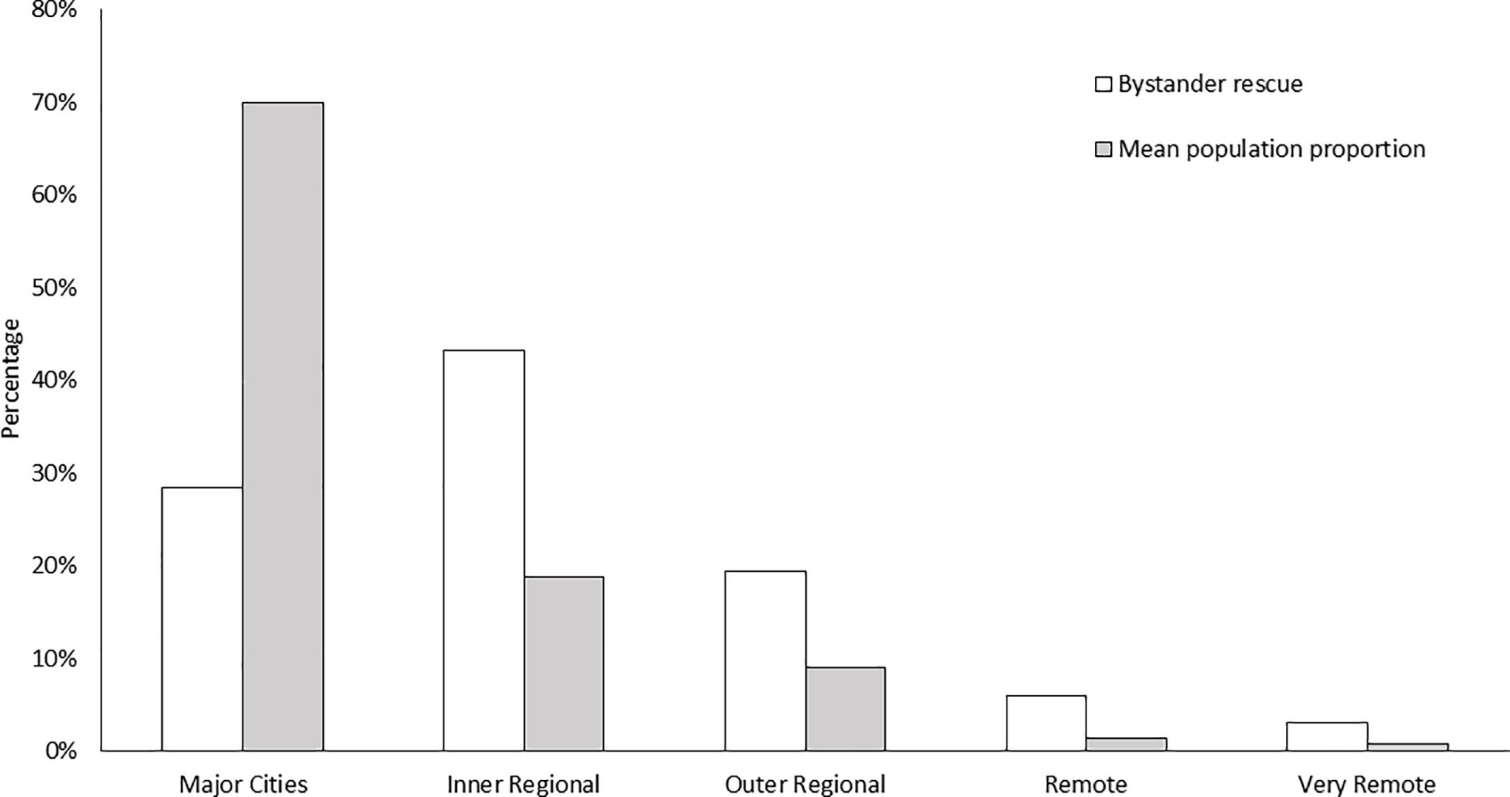
Remoteness classification of incident locations compared with mean population proportions (2004–19).

The location type where fatal bystander rescue incidents occurred differed (*Χ*^*2*^ = 9.421; p = 0.024) with most occurring at a beach (64%, n = 43), followed by rock/cliff locations (22%), and offshore waters (6%, n = 4). Differences also existed based on the remoteness classifications (*Χ*^*2*^ = 61.654; p < 0.001; Table 2; Fig 3). Most incidents occurred at inner regional locations (43%, n = 29), followed by major cities (28%, n = 19) and outer-regional areas (19%, n = 13). Only six incidents (7%) occurred in remote or very remote locations (Fig 3). Over three-quarters of bystander fatalities (78%, n = 52) occurred further than 1 km from lifesaving club and associated services, although this did not differ from expected proportions of non-rescue incidents (*Χ*^*2*^ = 3.563; p = 0.168; Table 2).

**Fig 4:**
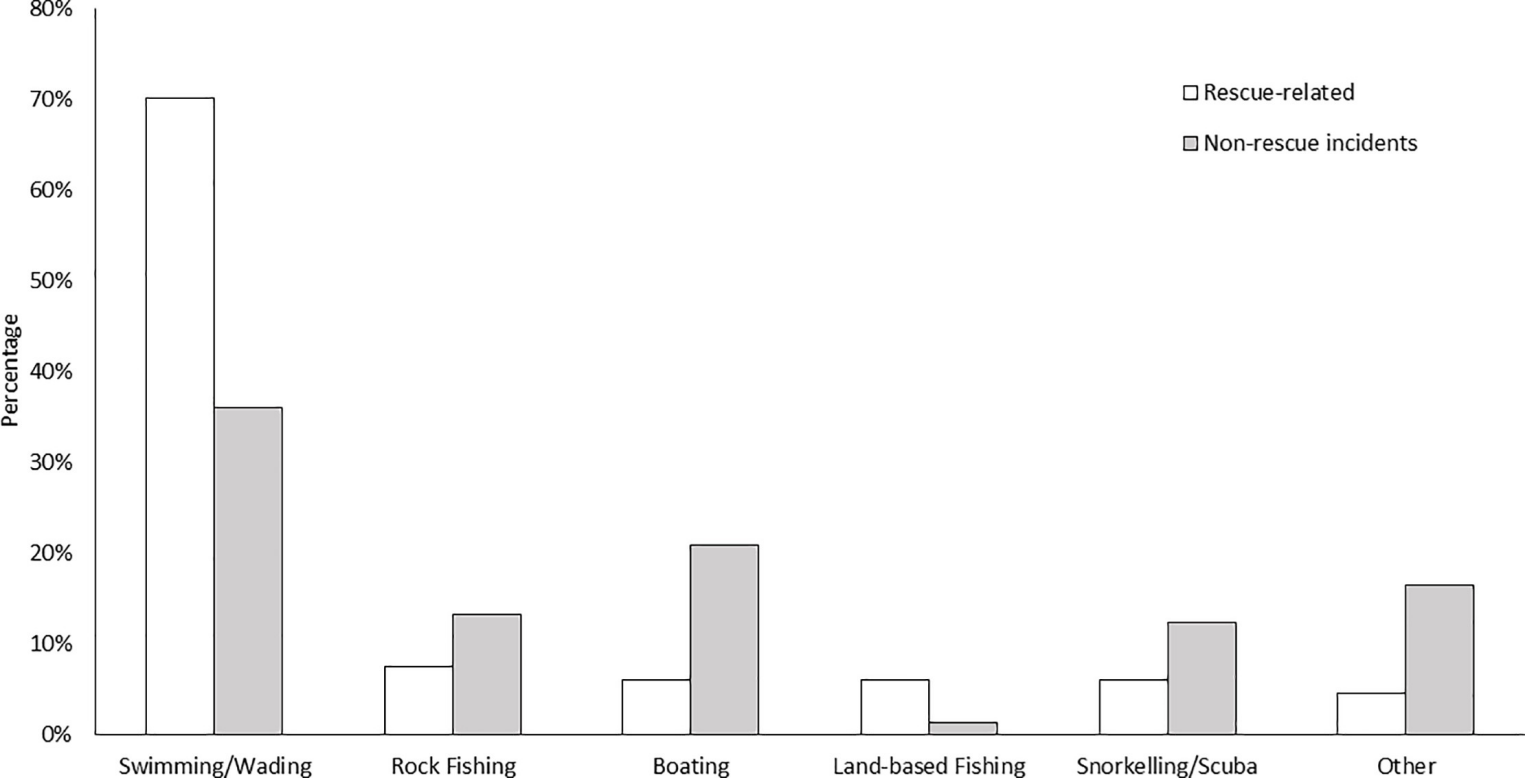
Activity participating in prior or incident compared to proportion of non-rescue incidents (2004–19).

The reason and activity victims were visiting the coast prior to fatal attempted rescues differed (*Χ*^*2*^ = 54.078; p < 0.001; Table 2; Fig 4) with swimming and wading (70%, n = 47) identified as the dominant activity prior to the incident, followed by rock fishing (7%, n = 5) and then boating (6%, n = 4).

Fatal bystander rescues occurred more than expected on weekend days than weekdays (*X*^*2*^ = 13.348; p < 0.001; Table 2) and the time of day that fatal bystander rescues occurred was also significant (*Χ*^*2*^ = 10.869; p = 0.004; Table 2). More incidents happened in the afternoon (72%, n = 48) compared to the morning (15%, n = 10) and evening (13%, n = 9). There were no bystander fatalities at night. The most common season for bystander rescuer fatalities was summer (45%, n = 30), followed by autumn (31%, n = 21) and spring (19%, n = 13; Table 2), although this did not differ from non-rescue proportions (*Χ*^*2*^ = 6.232; p = 0.101; Table 2).

Most bystander rescue victims were born in Australia (63%, n = 42) or Asia (19%, n = 13). These proportions differed from the mean population (*Χ*^*2*^ = 64.639; p = 0.015) with Asia- and Australian-born victims being proportionally higher and lower than the average population respectively (Table 2). Continent of residence for victims did not differ to population proportions (*Χ*^*2*^ = 1.078; p = 0.583; Table 2), with Australian residents accounting for 88% of fatal bystander rescues (n = 56) followed by Asian (9%, n = 6) and European residents (6%, n = 4).

The visitor status of fatal bystander victims differed to non-rescue incidents (*Χ*^*2*^ = 10.470; p = 0.033; Table 2), with incidents involving residents being lower that non-rescue incidents, while locals, interstate and intrastrate visitors were higher (Table 2). Over a third (35%, n = 22) of bystander victims were local residents who lived within 10 kilometres from the incident location, followed by intrastate visitors (31%, n = 19) and then interstate visitors (13%, n = 8; Table 2).

The relationship between the bystander rescuer and the rescuee differed (*Χ*^*2*^ = 69.537; p < 0.001; Table 2) with over two-thirds of fatal bystander incidents attempting to rescue a family member or loved one (69%, n = 46; Table 2). The next most common relationships were rescue attempts of a friend (15%, n = 10; Table 2) and a stranger (9%, n = 9; Table 1). A further three people died in other circumstances, including attempts to rescue an animal (combined into category ‘Other’, 7%, n = 5; Table 2). More incidents involved going to the aid of children (63%, n = 41) than adults (37%, n = 23) when the incident occurred (z = 41.0, p < 0.001; Table 2).

**Fig 5:**
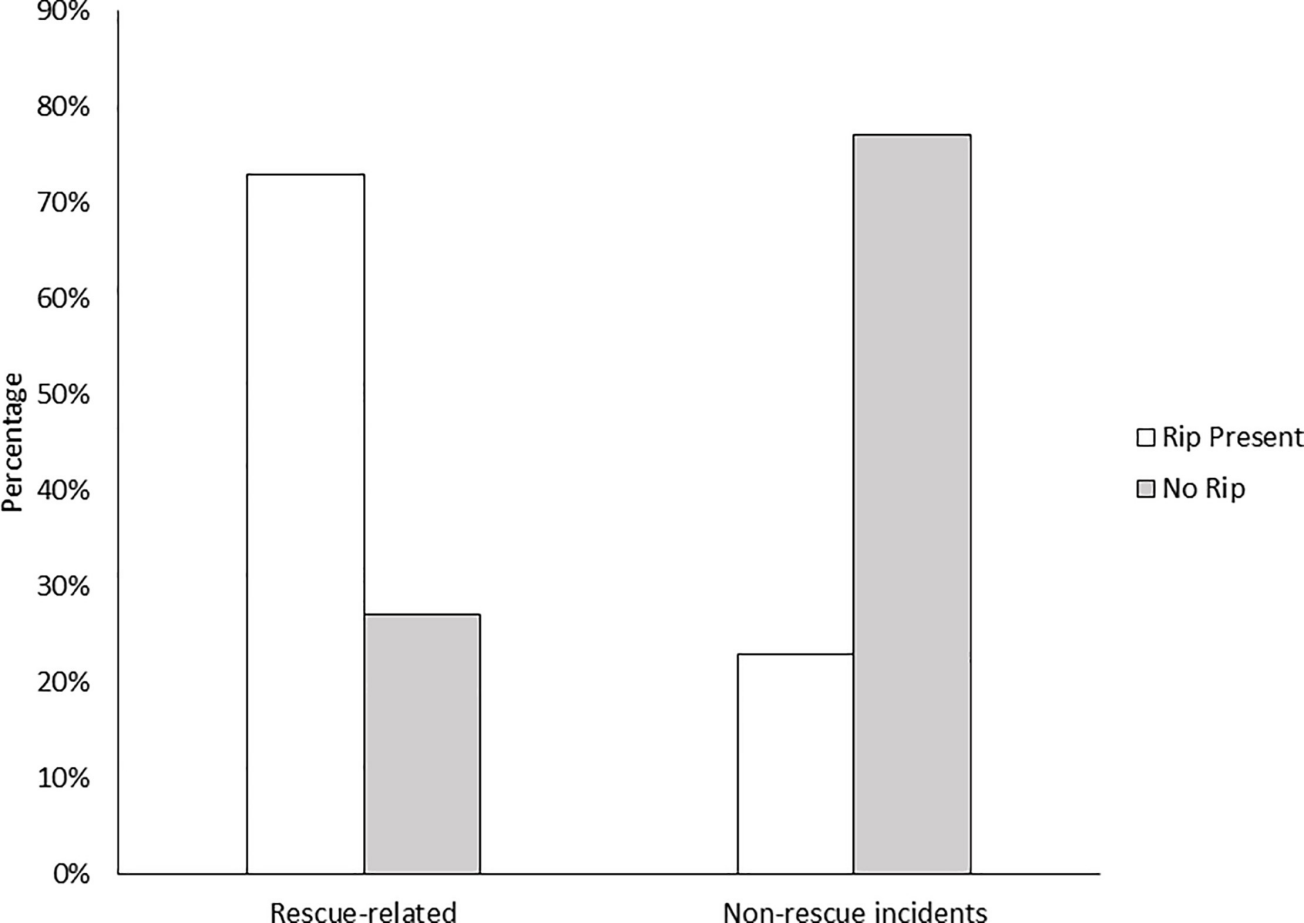
Presence of rip currents in fatal bystander rescues compared with non-rescue incidents.

The presence of rip currents was significantly higher for fatal bystander incidents when compared to other non-rescue incidents (73%; n = 46; *Χ*^*2*^ = 88.793; p < 0.001; Fig 5). Almost all incidents that occurred at a beach reported the presence of rip currents (93%, n = 40).

The overwhelming majority of bystander victims (97%, n = 65) did not use a flotation device when conducting their rescue (z = 64.000; p < 0.001; Table 2). Alcohol and/or drugs did not play a significant role in bystander incidents (*Χ*^*2*^ = 2.250; p = 0.134; Table 2) contributing to 15% (n = 10) of rescue-related fatalities.

## Discussion

Several studies have shown the important role that bystander rescuers play in saving lives in Australian coastal waters [9–11]. Unfortunately, this study has found that an average of 4.5 coastal bystander rescuer fatalities occur each year, a significant proportion of the annual average across all Australian waterways of five reported by Franklin, Peden [11]. Improved understanding of the characteristics of these coastal incidents is clearly warranted and is necessary for the development of future preventative strategies targeting bystander rescue scenarios in coastal waterways. As acknowledged by the World Health Organisation [4], prioritisation and planning of drowning prevention strategies begins with the identification of key factors from available data. This study provides incident and social profiles of the 67 fatal coastal bystander rescues in Australia between 2004–2019 with an aim to influence future evidence-driven targeted awareness campaigns.

### Incident characteristics of coastal bystander rescue fatalities

As described previously and documented in Table 2, the majority of coastal bystander rescuer fatality incidents in Australia occurred: i) in the state of New South Wales (NSW; 49%); ii) at beaches (64%); iii) in regional or remote areas (71%); iv) more than 1 km from the nearest lifesaving service (78%); v) during summer (45%); vi) in the afternoon (72%); vi) in the presence of rip currents (73%); and did not involve the use of flotation devices to assist rescue (97%). These findings are now discussed in context and compared primarily to recent studies of Franklin, Peden (11), who analysed bystander rescuer fatalities across all Australian waterways between 2006–2015, and Brander, Warton [10] who examined self-reported incidents of bystander rescues across all Australian waterways.

With 32% of the overall population NSW is Australia’s most populated state [24], but is over-represented in coastal drowning statistics in general [25]. This is likely to be due to its’ extensive coastline (1590 km), largely comprised of beaches (62%; Short 2007a) that are a major destination for both domestic and international visitors alike. Additionally, 85% (n = 757) of NSW beaches are classified as being open coast and wave dominated and considered to be hazardous [26], with many characterised by the presence of rip currents [27]. Rip currents are the major hazard on the Australian coast, contributing to an average of 21 confirmed drowning deaths per year [22, 23] and were associated with 93% of the beach-related bystander fatalities reported in this study (Table 2). Franklin, Peden [11] also found that beaches were the main location (54.9%) of fatal Australian bystander rescuer incidents and 90% of the self-reported coastal bystander rescues (76.5% of the total sample) in Brander, Warton [10] were associated with beaches. In comparison to beaches, rocks/cliffs were the second most common location of fatal bystander rescue incidents, but with a much lower incidence (22%; Table 2). This difference is likely a reflection of the more obvious perceived risks associated with attempting to rescue someone in rocky coast environments (which may involve jumping from height) compared to attempting rescues in more seemingly benign beach environments.

The fact that most incidents occurred in either regional or remote areas and more than 1 km from a lifesaving service is not surprising given that only a very small proportion of Australia’s estimated 11,000 mainland beaches are patrolled by lifeguard or lifesaving services, most of which are close to populated areas [25]. Additionally, most lifeguard and lifesaver services are seasonal and location specific with respect to patrol times. Furthermore, as shown by McKay, Brander [28] in the case of NSW at least, many coastal tourist accommodations are located in regional areas and are situated closest to hazardous surf beaches that are often unpatrolled. These findings are also consistent with Franklin, Peden [11] who reported 70.6% of bystander fatalities across Australian waterways occurring in rural locations and Brander et al. [10] who found that 67% of self-reported bystander rescues took place more than 1 km from lifeguard services.

The pattern of drowning deaths occurring in the afternoon is not uncommon [11, 29, RLSSA 30] and has previously been attributed to alcohol consumption [29] and warmer air temperatures [31]. This study did not find alcohol to play a significant role in fatal coastal bystander rescues. It is possible that the afternoon is a more social time of day when people may be more relaxed and/or fatigued. Higher fatigue levels, and/or increased distraction due to social interaction, may result in less reduced attention within a social cohort, or potential lapses in supervision by parents or guardians [32].

In the case of beaches, it is common for strong sea breezes to occur during the afternoon on warmer days, which may lead to the formation of wind waves and increased wave breaking activity [33, 34]. Wave breaking is the primary driving force behind rip currents [35] and it is possible that rip current activity may become more pronounced under these conditions. Indeed, most fatal bystander rescuer incidents occurred during summer (45%) and autumn (31%), seasons when water temperatures are also generally at their maximum providing favourable swimming conditions. These periods also include the summer holiday period in Australia from December to January as well as the extended school holiday period associated with Easter (generally in April). Of note, incidents occurred proportionally more on weekend days than weekdays which further reflects the Australian culture of recreating on the coast during leisure time. Franklin et al. [11] also found that the majority of bystander drowning deaths across all Australian waterways (45.1%) occurred during summer months.

Providing a flotation device to a drowning victim has been identified as a priority intervention that interrupts the drowning process and is crucial for mitigating effects on rescuer safety [36]. However, SLSA [37] and Brander, Warton [10] found that 60% and 63% of bystander rescuers respectively did not use a flotation device during their most recent rescue. Moran and Stanley [13] also reported that 30% of bystander rescuers did not think they would take a flotation device with them during a rescue scenario. In this study, only two rescue incidents involved the use of a flotation device, both of whom were conducted by (off duty) volunteer surf lifesavers with significant levels of water safety training. This supports the findings of Brander, Warton [10] who showed that bystander rescuers with prior water safety training are more likely to use a flotation device when attempting a rescue. Surfers also carry out a significant number of rescues on Australian beaches with the assistance of surf boards and other surf craft [9], which highlights the positive impact of having flotation when conducting a rescue.

### Social characteristics of coastal bystander rescue fatalities

In terms of characterising the coastal bystander fatality victims in Australia themselves, the results of this study (Table 2) have shown that the majority were: i) Australian residents (88%) born in Australia/Oceania (68%); ii) males (81%); iii) aged between 30–44 years old (36%); iv) visitors to the location (55%); v) either family (69%) or friends (15%) of the rescuee(s); and vi) attempting to rescue someone younger than 18 years old (64%).

Although most of the bystander rescue fatality incidents involved Australian residents, Asian-born victims (particularly those born in China and India) were at proportionally higher risks of fatal bystander rescue incidents (Table 2). Recently there has been increased attention regarding improving the understanding of water safety within culturally and linguistically diverse (CaLD) communities, who are often considered to be high-risk groups in terms of drowning [2]. Poor swimming ability, fewer opportunities to participate in water safety training (leading to less water safety skills), and less exposure to coastal hazards and environments (leading to lower risk perception) have previously been identified as key risk factors within Asian communities living in Australia [38].

It is well established that males are over-represented in coastal drowning deaths globally [22, 39–41] and bystander rescue events are no different in this regard [17, 18, 42, 43]. In the Australian context, the findings of this study are consistent with those of Franklin, Peden [11] who reported 82.4% of bystander rescuer fatalities across all waterways being male. This gender over-representation may be situational (i.e. more males were already in the water) or related to factors such as increased (or perceived) confidence in rescue abilities [42, 43] and greater willingness to conduct a rescue [13]. Unfortunately, the latter two factors rarely match with actual abilities or appropriate levels of water safety training [10, 11, 13, 43].

In terms of age, while most coastal bystander rescuer fatalities were aged between 30–44 years, 60% were aged between 18–44, which is consistent with the results of Franklin et al. [11] who reported 53% of bystander victims across all Australian waterways being aged between 25–44 years. Similarly, 25% of coastal bystander rescue fatality victims were aged 45–59 while Franklin, Peden [11] found that 30% of victims across all waterways were aged 45–64. To place these findings in perspective, it is important to consider two key aspects of bystander rescues: i) the relationship between the rescuer and the rescuee; and ii) the inherent impulsive nature of bystander rescues and their altruistic motivation, which is acknowledged to be especially challenging to address [5, 6, 13]. In both cases, bystander rescue scenarios are often highly emotive incidents [10], where the cost of not attempting a rescue outweighs the perceived risks with entering the water [44], which are often rarely considered or understood [6, 18].

The importance of the relationship(s) between rescuer and rescuee is evident by the fact that 69% and 15% of coastal bystander rescuers died while going to the aid of a family member or friend respectively. This is consistent with Franklin, Peden [11] who found that 51% of bystander rescuer fatalities across all Australian waterways involved family members, with 19.6% involving friends. In both cases, the age ranges of bystander rescuer victims reported above encompass life stages where many people are actively parenting or acting as carers. Indeed, most of the bystander rescuer victims in Australian coastal waterways (64%) were attempting to rescue children and adolescents less than 18 years old. In their study of self-reported bystander rescues across all waterways, Franklin, Peden [11] reported that 79% of bystander rescues involved rescuing someone less than 18 years old. These findings are consistent with the guardianship model defined in the aquatic victim instead of rescuer (AVIR) syndrome [6], where children are less likely to correctly assess and identify risks and parents/guardians will respond altruistically and impulsively. Similarly, a substantial amount of rescuees (36%; Table 1) were adults, of which almost half (43%; n = 10) were family and 35% (n = 8) were friends of the rescuer. This supports the extension of the AVIR syndrome to include all people known to the bystander rescuer [5].

In contrast, previous survey-based studies in Australia have shown that most self-reported bystander rescues involved rescuing strangers [9, 10]. However, these studies described successful rescues, where rescuers felt they had saved lives and were either more likely to have an appropriate level of prior water safety training [10], or, in the case of surfers, were already in the water [9]. Nevertheless, these findings highlight the importance of factors described in the AVIR model [5], such as training and situation/early response, which can lead to successful rescues–factors that may have been missing from the fatal coastal bystander rescues reported in this study.

It is interesting to note that this study found that 69% of coastal bystander rescuers died while going to the aid of a family member, compared to 51% reported by Franklin, Peden [11] across all Australian waterways. This difference may reflect the popularity of beaches as a family-oriented location, particularly during holiday periods. This notion is supported by the fact that 51% of bystander rescuer fatality victims were visitors (being either intrastate, interstate, or international; Table 1) during the summer and autumn months, which, as noted previously, encompass the Australian Christmas and extended school holiday period and the Easter school holidays (generally two weeks in April). This finding supports the hazardous nature of unfamiliar environments [e.g. 6, 45–47], which has been suggested to increase with distance or unfamiliarity [48] and is a common theme across injury prevention [e.g. 49].

However, 35% and 10% of bystander rescue victims reported in this study were either defined as locals (living within 10 km of the incident location) or residents (between 10–50 km) respectively. Raising awareness levels of local environmental hazards in visitors is considered as an avenue to prevent incidents in unfamiliar environments [47], although this has also been suggested to lead to an underestimation of the local drowning risks [50]. We further this to propose the concept of a ‘locality effect’, where greater exposure to nearby hazards puts locals at greater risk of being involved in a fatal bystander rescue event where familiarity of local environments and associated hazards may lead to complacency and therefore an underestimation of associated risks [50].

### Implications

Bystander rescues can be highly emotive situations, often involving direct family intervention, and while bystander rescuer drowning deaths may only account for a small number of the 15-year average of 110 coastal drowning deaths per year in Australia [25], they are particularly important as ‘anyone’ potentially represents a potential bystander rescuer. Presently there is no targeted safety intervention or education campaign that specifically deals with the issue of bystander rescues in Australia. This study has provided a valuable profile of the characteristics of bystander rescuer fatalities in Australian coastal waterways that allows for specific recommendations to be made in order to reduce the future occurrence of these incidents.

Future safety interventions should primarily target beach locations, particularly those in popular coastal holiday locations in regional areas, where lifeguard and lifesaver services may not always be present. As suggested by Pearn and Franklin [51] and Brander, Warton [10], bystander rescue safety messaging needs to emphasise the importance of having access to a flotation device (e.g. a boogie board) while visiting a beach (or knowing where to find one). Rip currents play a significant role in bystander rescuer fatalities on Australian beaches and future interventions should aim to improve beachgoer awareness and recognition of this hazard.

Our results indicate that future interventions should target males, but should also be linked to emotionally driven responses from parents, family members and carers, which supports the call for increased access to survival skill training for bystanders [3, 11] and the concept of having clearly designated child supervisors [32]. Bystander rescue prevention strategies should also focus on both locals and domestic visitors, with an emphasis at common holiday locations in regional areas. There is evidence to support the need for targeted education, training and awareness campaigns for the CaLD community with appropriate messaging designed for high-risk communities from specific ethnic backgrounds especially involving swimming ability and risks associated with recreating on the coast.

General strategies for drowning prevention are typically outlined as involving education, design, and policy [5]. However, in terms of drowning prevention, bystander rescue events are relatively complex. Education that trains bystanders how to conduct a safe rescue (preferably non-contact) with regular refresher courses to maintain skills has been highlighted as a key strategy to reduce the number of fatal rescues [3, 5, 6, 11]. However, implementing and maintaining water safety training levels across the population, and even sub-sections of the population is a logistically daunting task. There is also little evidence to suggest that interventions involving safety signage and brochures related to beach safety messaging are necessarily effective [52–54]. The question then becomes–what should the nature of future bystander rescue safety interventions involve?

Given the emotive nature involving bystander rescue fatalities, it is possible that Public Service Announcements, or billboard advertisements, which involve confronting imagery and messaging may be adopted that prove effective. Alternatively, messaging may be communicated through these means that simply inform coastal visitors with the appropriate responses when faced with an emergency (e.g. don’t rush in, seek help, look for a flotation device). These transformative approaches, however, require a higher level of ownership where the onus is on the citizen to take responsibility for their own safety, in what is often an impulsive situation [6]. In this vein, Surf Life Saving Australia recently developed a national beach safety awareness campaign involving the Think Line [23]–which encourages people to ‘Stop, Look and Plan’ before and during their visit to the coast. While the focus of this campaign has been initially on the rip current hazard, it could easily be extended to include other aspects of beach safety, including bystander rescues. The motivation for this campaign was to instil an intrinsic safety message for all beachgoers.

However, before any bystander rescuer safety intervention is adopted, it is critical that future research examine the potential effectiveness of various approaches so that any future campaign, slogan, or intervention material is evidence-based. Pearn and Franklin [5] emphasised the importance of understanding human behaviour in relation to bystander rescue situations. Future research into the psychology of these incidents, which could best be obtained via interviews with bystander rescuers, is also vital for the development of effective preventative strategies.

### Conclusions

This study represents the first data review of coastal bystander rescuer fatalities in Australia and provides a valuable profile of these incidents as well as suggestions on how to approach future safety interventions. While it is evident from both this study and previous research that a multi-faceted approach is necessary to address the problem from an intervention perspective, it is also the case that bystander rescue situations are particularly complex compared to other aspects of drowning prevention due to their associated instinctive, impulsive and altruistic human behaviour. Future research therefore needs to focus on the psychology of the bystander rescuer in order to truly engage in future safety interventions that will be effective.
